# Ovatodiolide Inhibits Breast Cancer Stem/Progenitor Cells through SMURF2-Mediated Downregulation of Hsp27

**DOI:** 10.3390/toxins8050127

**Published:** 2016-04-28

**Authors:** Kuan-Ta Lu, Bing-Yen Wang, Wan-Yu Chi, Ju Chang-Chien, Jiann-Jou Yang, Hsueh-Te Lee, Yew-Min Tzeng, Wen-Wei Chang

**Affiliations:** 1 Department of Anesthesiology, Changhua Christian Hospital, Changhua 500, Taiwan; 97343@cch.org.tw; 2 Division of Thoracic Surgery, Department of Surgery, Changhua Christian Hospital, Changhua 500, Taiwan; 156283@cch.org.tw; 3 School of Medicine, Kaohsiung Medical University, Kaohsiung 807, Taiwan; 4 Institute of Genomics and Bioinformatics, National Chung Hsing University, Taichung 402, Taiwan; 5 School of Biomedical Sciences, Chung Shan Medical University, Taichung 40201, Taiwan; zx82704@yahoo.com.tw (W.-Y.C.); swan1204@hotmail.com (J.C.-C.); jiannjou@csmu.edu.tw (J.-J.Y.); 6 Institute of Microbiology & Immunology, Chung Shan Medical University, Taichung 40201, Taiwan; 7 Department of Medical Research, Chung Shan Medical University Hospital, Taichung 40201, Taiwan; 8 Institute of Anatomy and Cell Biology, School of Medicine, National Yang Ming University, Taipei 11221, Taiwan; incubator.lee@ym.edu.tw; 9 Center for General Education, National Taitung University, Taitung 95092, Taiwan; 10 Department of Appiled Chemistry, Chaoyang University of Technology, Taichung 41349, Taiwan

**Keywords:** ovatodiolide, cancer stem/progenitor cells, Hsp27, SMURF2

## Abstract

Cancer stem/progenitor cells (CSCs) are a subpopulation of cancer cells involved in tumor initiation, resistance to therapy and metastasis. Targeting CSCs has been considered as the key for successful cancer therapy. Ovatodiolide (Ova) is a macrocyclic diterpenoid compound isolated from *Anisomeles indica* (L.) Kuntze with anti-cancer activity. Here we used two human breast cancer cell lines (AS-B145 and BT-474) to examine the effect of Ova on breast CSCs. We first discovered that Ova displayed an anti-proliferation activity in these two breast cancer cells. Ova also inhibited the self-renewal capability of breast CSCs (BCSCs) which was determined by mammosphere assay. Ova dose-dependently downregulated the expression of stemness genes, octamer-binding transcription factor 4 (Oct4) and Nanog, as well as heat shock protein 27 (Hsp27), but upregulated SMAD ubiquitin regulatory factor 2 (SMURF2) in mammosphere cells derived from AS-B145 or BT-474. Overexpression of Hsp27 or knockdown of SMURF2 in AS-B145 cells diminished the therapeutic effect of ovatodiolide in the suppression of mammosphere formation. In summary, our data reveal that Ova displays an anti-CSC activity through SMURF2-mediated downregulation of Hsp27. Ova could be further developed as an anti-CSC agent in the treatment of breast cancer.

## 1. Introduction

Cancer stem/progenitor cells (CSCs) have been described for decades and these particular cancer cells have been reported to be involved in tumor initiation, resistance to chemotherapy or radiotherapy, and metastasis [1,2]. Breast CSCs were first identified by Al-Hajj *et al.* with the marker of CD24-CD44+ [3]. Ginestier *et al.* later reported that breast cancer cells with high intracellular aldehyde dehydrogenase (ALDH) activity also represented the population of BCSCs [4]. In addition to cell surface markers or intracellular enzyme activity, BCSCs could be enriched with a cultivation method of the mammosphere, a clump of cancer cells with stem/progenitor cell properties [5]. The drug screening results from tumorsphere assay have been reported to be more translatable than those from the 2-dimensional adherent condition [6,7,8,9]. Targeting CSCs is considered as a key for successful treatment in cancer [2,10].

Heat shock proteins (Hsps) are a group of stress-induced proteins with a molecular chaperone function to maintain or correct the structure of intracellular proteins [11]. Several Hsps have been reported to be overexpressed in cancers, such as Hsp90 and Hsp27 [12]. Hsp27 belongs to small Hsps and its high expression in breast cancer tissues has been reported to be associated with lymph node metastasis [13]. We previously discovered that Hsp27 was upregulated in ALDH+ BCSCs [14]. Knockdown of Hsp27 in ALDH+ BCSCs resulted in the inhibition of epithelial-mesenchymal transition (EMT) and tumorigenicity [14]. We also demonstrated that the phosphorylation of Hsp27 was involved in the epidermal growth factor (EGF)-induced vasculogenic mimicry activity of BCSCs [15]. Agents that display the activity in Hsp27 inhibition are potentially being developed as anti-breast cancer drugs.

Ovatodiolide (Ova) is a macrocyclic diterpenoid compound extracted from *Anisomeles indica* (L.) Kuntze [16] with activities of anti-inflammation [17], anti–*Helicobacter pylori* [18], dermatological whitening [19], and anti-neoplasm [20,21,22,23]. Here we report that Ova displays an anti-CSC activity in breast cancer. Ova dose-dependently suppressed the self-renewal property of BCSCs and inhibited the expression of stemness genes, such as octamer-binding transcription factor 4 (Oct4) and Nanog. We further demonstrated that the anti-BCSC activity of Ova was mediated by the downregulation of Hsp27 through the induction of SMAD-specific E3 ubiquitin protein ligase 2 (SMURF2).

## 2. Results

### 2.1. Ovatodiolide Inhibited Self-Renewal Capability of BCSCs

**Figure 1:**
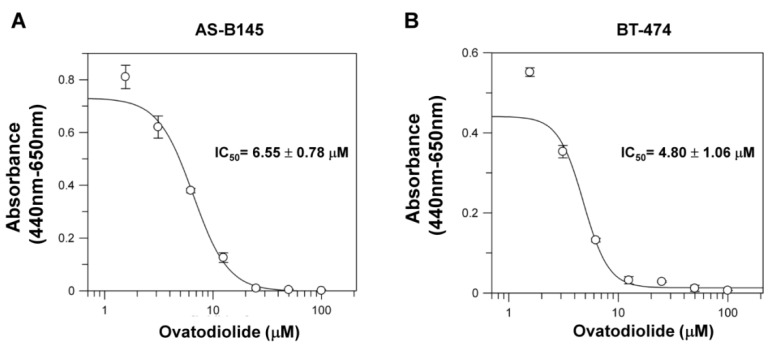
The cytotoxic effect of ovatodiolide in human breast cancer cells. AS-B145 (**A**) or BT-474 (**B**) cells were seeded in a 96-well plate and treated with a different concentration of ovatodiolide (0, 1.625, 3.125, 6.25, 12.5, 25, 50, 100 μM) for 72 h (*n* = 4 for each concentration). Cell proliferation was determined by WST-1 reagent and the IC50 value was calculated by GraFit software. The experiments were repeated two times and results from a representative experiment were presented.

**Figure 2:**
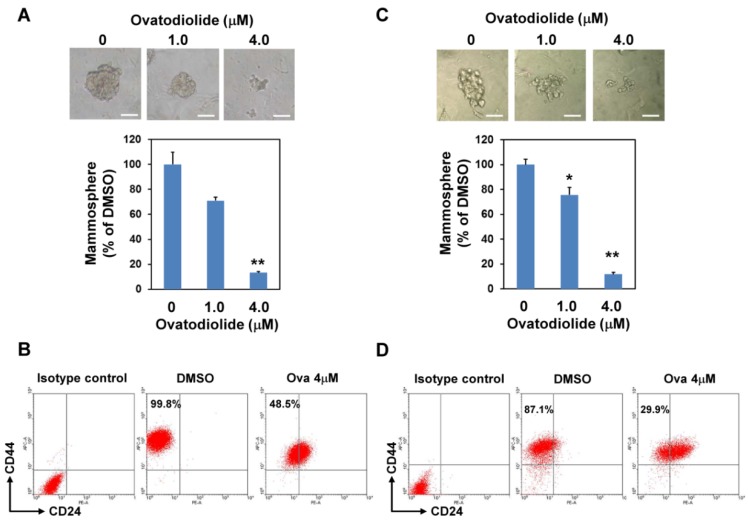
Ovatodiolide suppresses the self-renewal property of BCSCs. AS-B145 (**A**) or BT-474 (**B**) cells were seeded into ultralow attachment in a six-well plate under 0.1% DMSO or different concentrations of ovatodiolide (1 or 4 μM) for seven days and the formed primary mammospheres were collected and dissociated into a single cell suspension. The same number of dissociated primary sphere cells was used to evaluate the effect of ovatodiolide on the self-renewal property of BCSCs by secondary mammosphere formation without treatment of ovatodiolide (*n* = 3 for each treatment). The experiments were repeated two times and results from a representative experiment were presented. Data were presented as relative percentage of DMSO control. Scale bar = 50 μm. *, *p* < 0.05; **, *p* < 0.01. The mammosphere cells of AS-B145 (**C**) or BT-474 (**D**) were harvested and dissociated into a single-cell suspension. CD24-CD44+ cells were analyzed by flow cytometry.

We first determined the effect of Ova in cell proliferation of breast cancer cells. With the WST-1 assay, Ova displayed an anti-proliferation effect on AS-B145 and BT-474 human breast cancer cells and the IC_50_ value was 6.55 ± 0.78 μM (Figure 1A) and 4.80 ± 1.06 μM (Figure 1B) for AS-B145 and BT-474, respectively. Mammosphere cultivation is a method to enrich and to analyze the self-renewal capability of BCSCs [8]. We next applied the mammosphere assay to evaluate the anti-self-renewal activity of Ova. AS-B145 or BT-474 cells were cultivated into primary mammospheres in the presence of Ova at the concentration of 1 or 4 μM, which was below the IC_50_ value in the proliferation inhibition effect, and the self-renewal capability of primary spheres was determined by the formation of secondary mammospheres without Ova treatment. As shown in Figure 2, Ova dose-dependently inhibited the formation of the secondary mammosphere of AS-B145 (Figure 2A) and BT-474 (Figure 2B). The CD24-CD44+ BCSCs were also analyzed in AS-B145 or BT-474 sphere cells. After treatment of Ova at a concentration of 4 μM, the population of CD24-CD44+ cells in mammospheres of AS-B145 (Figure 2C) or BT-474 (Figure 2D) was decreased (from 99.8% to 48.5% for AS-B145 and from 87.1% to 29.9% for BT-474). From these results, Ova displayed an anti-self-renewal activity in BCSCs.

### 2.2. Ovatodiolide Downregulated the Expression of Stemness Genes and Hsp27 but Upregulated SMURF2 Expression

**Figure 3:**
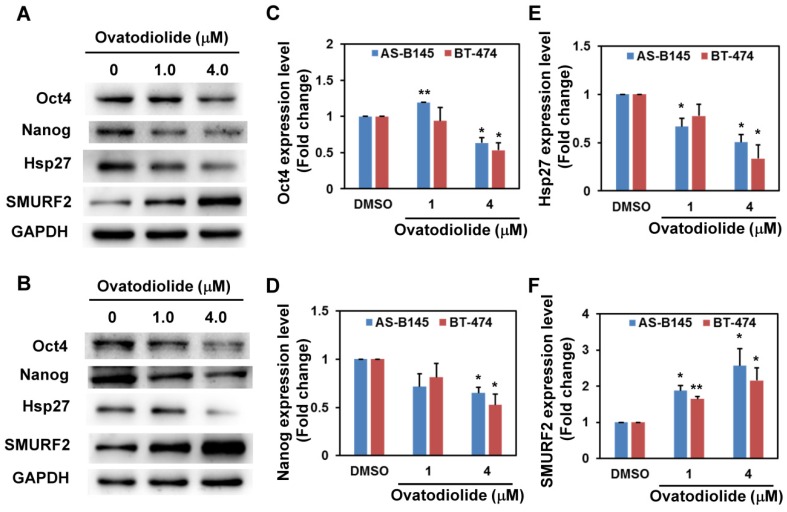
The change of protein expression in ovatodiolide-treated BCSCs. BCSCs were first enriched by primary mammosphere cultivation from AS-B145 (**A**) or BT-474 (**B**), dissociated into a single-cell suspension, and treated with different concentrations of ovatodiolide (0, 1, 4 μM) for 72 h (*n* = 2 for each treatment). The expressions of Oct4, Nanog, Hsp27 and SMURF2 were determined by Western blot. The quantification results of Oct4 (**C**), Nanog (**D**), Hsp27 (**E**) and SMURF2 (**F**) were determined by Image J software. The experiments were repeated three times and results from two representative experiments were used for quantifications. * *p* < 0.05; ** *p* < 0.01.

We next examined the effect of Ova on the expression of stemness genes. With Western blot analysis, Ova dose-dependently inhibited the expression of Oct4 (Figure 3C) and Nanog (Figure 3D) in mammosphere cells derived from AS-B145 (Figure 3A) or BT-474 (Figure 3B) and the inhibitory effect was significantly observed at a concentration of 4 μM. We previously demonstrated that Hsp27 regulated the self-renewal and tumorigenicity of BCSCs through modulating EMT and NF-κB activity [14]. The effect of Ova in Hsp27 expression in mammosphere cells was examined. As shown in Figure 3, Ova dose-dependently downregulated Hsp27 expression (Figure 3E) in mammosphere cells derived from AS-B145 (Figure 3A) or BT-474 (Figure 3B). However, the mRNA expression of Hsp27 in mammosphere cells of AS-B145 or BT-474 was not inhibited by Ova treatment (Figure S1). A previous report indicated that SMURF2 mediated the ubiquitin-dependent degradation of Hsp27 in A549 lung cancer cells [24]. We further examined the expression of SMURF2 in mammosphere cells after Ova treatment and results revealed that Ova dose-dependently upregulated SMURF2 expression (Figure 3F) in AS-B145 (Figure 3A) or BT-474 (Figure 3B).

### 2.3. Overexpression of Hsp27 or Knockdown of SMURF2 Alleviated the Inhibitory Effect of Ovatodiolide

**Figure 4:**
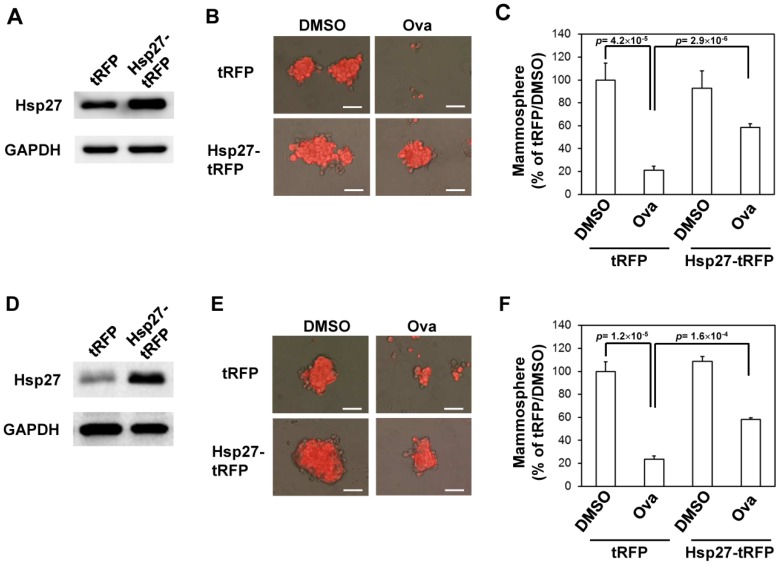
Overexpression of Hsp27 diminishes the inhibitory effect of ovatodiolide on the self-renewal capability of BCSCs. AS-B145 or BT-474 cells were transduced with tRFP or Hsp27-tRFP lentivirus and selected by 20 μg/mL blasticidin S for one week. The overexpression of Hsp27 was confirmed by Western blot ((**A**) for AS-B145 and (**D**) for BT-474). BCSCs were first enriched by primary mammosphere cultivation from tRFP- or Hsp27-overexpressed cells, dissociated into a single-cell suspension, and underwent secondary mammosphere formation under treatment with 4 μM ovatodiolide (Ova) or 0.1% DMSO (*n* = 3 for each treatment). Formed mammospheres were pictured ((**B**) for AS-B145 and (**E**) for BT-474) and were counted at Day 7 and displayed as the relative percentage of the DMSO group ((**C**) for AS-B145 and (**F**) for BT-474). The experiments were repeated two times and results from a representative experiment were presented. Scale bar = 50 μm.

**Figure 5:**
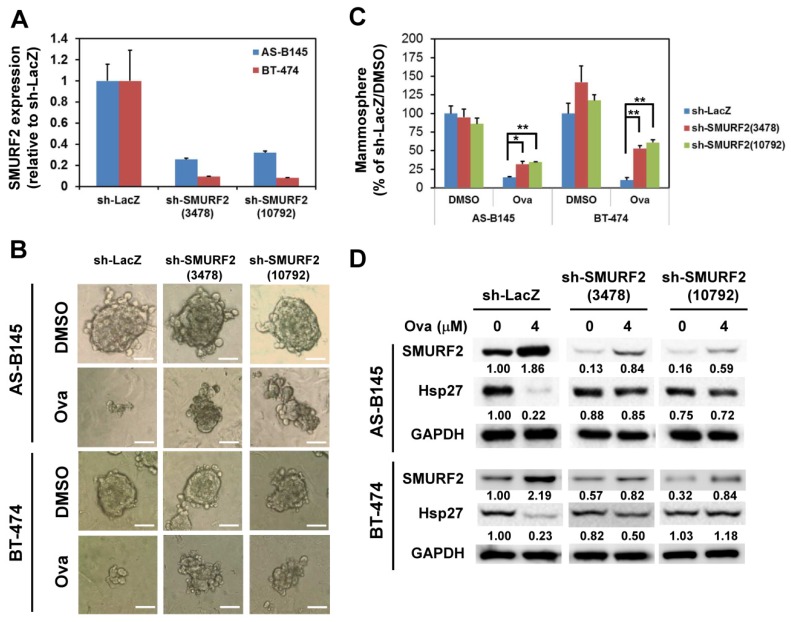
Knockdown of SMURF2 alleviates the suppressive effect of ovatodiolide on the self-renewal capability of BCSCs. AS-B145 or BT-474 cells were transduced with shellacs, sh-SMURF2(3478), or sh-SMURF2(10792) carrying lentivirus and selected by 2 μg/mL puromycin for three days. The knockdown efficiency was determined by qRT-PCR (**A**). After puromycin selection, the surviving cells were first cultured for primary mammosphere formation. The self-renewal capability of primary mammospheres under 4 μM ovatodiolide (Ova) or 0.1% DMSO was determined by the formation of secondary mammospheres (*n* = 3 for each treatment). Formed secondary mammospheres were pictured (**B**) and were counted at Day 7 and displayed as the relative percentage of the DMSO-treated sh-LacZ group (**C**). Scale bar = 50 μm. *, *p* < 0.05; **, *p* < 0.01. The expression of Hsp27 or SMURF2 was further determined by Western blot (**D**). The experiments were repeated two times and results from a representative experiment were presented.

We next examined if overexpression of Hsp27 could alleviate the inhibitory effect of Ova on the mammosphere formation capability of AS-B145 cells. Overexpression of Hsp27 in AS-B145 or BT-474 cells was performed by lentivirus-mediated gene delivery and confirmed by Western blot (Figure 4A). Exogenous Hsp27 expression in AS-B145 (Figure 4B,C) or BT-474 (Figure 4D,E) significantly diminished the suppressive activity of Ova on mammosphere formation at a concentration of 4 μM (*p* = 2.9 × 10^−6^ for AS-B145 and *p* = 1.6 ×10^−4^ for BT-474). We further performed the knockdown of SMURF2 in AS-B145 or BT-474 cells by lentivirus delivery of two independent shRNA clones and both clones efficiently knocked down the mRNA expression of SMURF2 (Figure 5A). The inhibitory effect of Ova in mammosphere formation was alleviated in both sh-SMURF2 clone-transduced AS-B145 or BT-474 cells (Figure 5B,C). We also analyzed the protein expression of Hsp27 and SMURF2 in shRNA-carrying lentviruse-transduced AS-B145 or BT-474 mammosphere cells after Ova treatment. As shown in Figure 5D, these two sh-SMURF2 lentiviruses efficiently knocked down SMURF2 protein expression in AS-B145 and BT-474 mammosphere cells and alleviated the inhibitory effect of Ova in Hsp27 protein expression (Figure 5D). These results suggest the suppressive effect of Ova in the self-renewal of BCSCs through the SMURF2-mediated downregulation of Hsp27 expression.

## 3. Discussion

Recently, Bamodu *et al.* reported that Ova sensitized breast cancer cells to doxorubicin and inhibited their CSC activity [22]. Here we also demonstrated the anti-self-renewal activity of Ova in BCSCs (Figure 2). Hsp27 has been reported to be involved in the chemoresistant property of CSCs [25,26]. Knockdown of Hsp27 increased the susceptibility of Herceptin-resistant SKBR3 cells to Herceptin [27]. The survival role of Hsp27 has been reported to be mediated through the activation of Akt [28] and inhibition of Bax activation [29]. Together with our findings, the sensitization activity of Ova to chemotherapy drugs in breast cancer cells may also be mediated through the downregulation of Hsp27.

Lin *et al.* previously demonstrated that Ova inhibited the metastatic ability of MDA-MB-231 breast cancer cells through suppression of p38 mitogen-activated protein kinase (MAPK) and phosphatidylinositol 3-kinase/Akt activation [21]. p38-MAPK has been known as one of the upstream kinases in Hsp27 phosphorylation [30]. We previously reported that the activation of p38 MAPK and Hsp27 phosphorylation was upregulated in BCSCs [14]. The knockdown of Hsp27 in BCSCs resulted in the suppression of the EMT features [14]. Hsp27 has been demonstrated to be involved in transforming growth factor β–induced EMT through the maintenance of Snail expression [31]. The emergence of CSCs could be a result of EMT [32]. Here we reported the inhibitory activity of Ova on the Hsp27 expression of BCSCs (Figure 3). The anti-CSC activity of Ova may be mediated through its inhibitory activity in EMT but remains to be further investigated. Ho *et al.* reported that Ova inhibited β-catenin signaling in renal cell carcinoma [23]. Hsp27 has been reported to interact with cytoplasmic β-catenin in breast cancer cells and clinical specimens [33]. It is worth investigating the effect of Ova on the Hsp27–β-catenin interaction in BCSCs.

## 4. Conclusions

In conclusion, we observed that Ova displayed anti-self-renewal activity in the mammopshere formation of human breast cancer cells as well as lead to the downregulation of stemness genes such as Oct4 and Nanog. The anti-BCSC activity of Ova was mediated through SMURF2-mediated downegulation of Hsp27. These findings reveal the potential of Ova in breast cancer treatment.

## 5. Materials and Methods

### 5.1. Reagents and Antibodies

Ovatodiolide was prepared as the previous report [17]. WST-1 cell proliferation reagent was purchased from Roche Life Science (Indianapolis, IN, USA). Polyclonal rabbit anti-Oct4 (CST-2750) or anti-Nanog (CST-3580) antibodies were purchased from Cell Signaling Technology, Inc. (Danvers, MA, USA). Polyclonal rabbit anti-Hsp27 antibody (ADI-SPA-803) was purchased from Enzo Life Sciences, Inc. (Farmingdale, NY, USA). Polyclonal rabbit anti-SMURF2 antibody (GTX110487) was purchased from GeneTex International Corporation (Hsinchu City, Taiwan). Peroxidase conjugated ployclonal goat anti-rabbit IgG secondary antibody (AP132P) was purchased from Merck Millipore (Temecula, CA, USA).

### 5.2. Cell Culture and Cytotoxicity Assay

AS-B145 human breast cancer cells were established from a female breast cancer specimen as our previous report [14,34] and maintained in MEMα (Gibco, Invitrogen Corporation, Carlsbad, CA, USA) supplemented with 10% fetal bovine serum (FBS, Gibco) and 5 μg/mL insulin (Sigma-Aldrich, St. Louis, MO, USA) at 37 °C, 5% CO_2_ incubator. BT-474 human breast cancer cells were obtained from The Bioresource Collection and Research Center in Taiwan (Hsinchu City, Taiwan) and cultured in DMEM/F12 (Gibco) supplemented with 10% FBS. For determination of cytotoxic effect of Ova, AS-B145 or BT-474 cells were seeded as 2 × 10^4^ cells/well in 96-well plates in presence of different concentration of Ova and cultured at 37 °C for 72 h. The proliferation/survival of cells was determined by adding WST-1 reagent and measured the absorbance of 440 and 650 nm wavelength. IC_50_ values were calculated by GraFit software (version 7, Erithacus Software Ltd., West Sussex, UK, 2012).

### 5.3. Mammosphere Cultivation

For primary mammosphere formation, AS-B145 or BT-474 cells were suspended as 1 × 10^4^/well/2 mL and cultured in ultralow attachment 6-well-plates (Corning, Lowell, MA, USA) in DMEM/F12 medium supplemented with 0.4% bovine serum albumin (Sigma), 20 ng/mL EGF (PeproTech, Rocky Hill, NJ, USA) , 20 ng/mL basic fibroblast growth factor (Sino Biological Inc., Beijing, China), 4 μg/mL heparin (Sigma-Aldrich), 5 μg/mL insulin, 1 μM hydrocortisone (Sigma-Aldrich), and 1X B27 supplement (Gibco). After 7 days, primary mammospheres with diameter larger than 50 μm were collected with 70 μm cell strainer (BD Biosciences, San Jose, CA, USA) and dissociated into single cell suspension with HyQTase (GE Healthcare Life Sciences HyClone Laboratories, Logan, UT, USA) for secondary mammosphere formation at a cell density as 2500 cells/well/2mL for further seven days. The form secondary mammospheres were pictured and counted with an inverted microscopy (AE30, Motic Electric Group Co., Ltd., Xiamen, China).

### 5.4. Analysis of CD24-CD44+ Cells

Mammosphere cells were dissociated into single cell suspension with HyQTase solution. 1 × 10^5^ dissociated cells were stained by anti-human CD24-PE (Cat. No. 555428, BD Biosciences) and anti-human CD44-APC (Cat. No. 559942, BD Biosciences) conjugated antibodies in staining buffer (PBS containing 1% FBS and 0.05% NaN_3_) on ice for 30 min. PE conjugated mouse IgG2a κ (Cat. No. 555574, BD Biosciences) and APC conjugated mouse IgG2b κ (Cat. No. 555745, BD Biosciences) antibodies were used as isotype controls. After washing with 2 ml PBS/0.01% NaN_3_, the fluorescence signals of stained cells were acquired with FACSAria^TM^ flow cytometer (BD Biosciences) and the data were further analyzed by WinMDI software (version 2.9, The Scripps Research Institute, La Jolla, CA, USA, 2000).

### 5.5. Western Blot

Mammospheres were collected by centrifugation and lysed in M-PER Mammalian Protein Extraction Reagent (Pirece Thermo Fisher Scientific Inc., Waltham, MA, USA). Then 20 μg of total protein were separated by SDS-PAGE and transferred onto PVDF membrane (Immobilon-P, Merck Millipore). The membrane was then blocked with 5% skimmed milk (Sigma-Aldrich, St. Louis, MO, USA) dissolved in Tris buffered slaine (Sigma-Aldrich) containing 0.05% Tween-20 (Sigma-Aldrich) (TBS-T) at room temperature for 1 h followed by incubation with primary antibodies at 4 °C overnight. After washing with TBS-T, the membrane was then incubated with peroxidase conjugated secondary antibodies (PerkinElmer, Waltham, MA, USA) at room temperature for one hour. The signals were developed by ECL-plus chemiluminescence substrate (PerkinElmer) and captured using a Luminescent Image Analyzer (Fusion SOLO, Vilber Lourmat, Marne-la-Vallée, France). The band intensity was quantified using ImageJ software (version 1.48a, NIH, Bethesda. MA, USA, 2013).

### 5.6. Lentivirus Production and Transduction

Hsp27 cDNA was amplified from pDsRed-Hsp27 [14] and cloned into pLAS5w.Pbsd-L-tRFP lentiviral plasmid (obtained from the National RNAi Core Facility at the Institute of Molecular Biology, Academia Sinica, Taipei, Taiwan) with following primers:

AfeI-Hsp27-F: 5’-AATAGCGCTATGACCGAGCGCCGCGTCCCC-3’
Hsp27-EcoRI-R: 5’-CGCGAATTCTTACTTGGCGGCAGTCTCATC-3’

The amplified DNA fragments and pLAS5w.Pbsd-L-tRFP plasmid were digested with AfeI and EcoRI restriction enzymes followed by ligation and transformation into Stbl3 competent cells (Life Technologies, Carlsbad, CA, USA). Lentiviral shRNA plasmids (TRCN00023122 for sh-LacZ, TRCN0000003478 for sh-SMURF2(3478), or TRCN0000010792 for sh-SMURF2(10792)) were also obtained from the National RNAi Core Facility. For lentivirus production, individual lentiviral plasmid was mixed with pCMV-ΔR8.91 and pMD.G plasmids as a ratio of 1:0.9:0.1 and transfected into 293T cells by GenJet^TM^ Transfection Reagent (SignaGen Laboratories, Ijamsville, MD, USA). Cells were transduced with lentivirus as MOI = 1 in presence of 8 μg/mL polybrene (Sigma-Aldrich) for 24 h and selected by blasticidin S (for Hsp27) or puromycin (for sh-LacZ or sh-SMURF2 clones) (TOKU-E Company, Bellingham, WA, USA).

### 5.7. Quantitative Reverse Transcription Polymerase Chain Reaction (qRT-PCR)

Total RNA was extracted by Quick-RNA^TM^ MiniPrep Kit (Zymo Research Corporation, Irvine, CA, USA). Then 1 μg of extracted RNA was used for cDNA synthesis with random hexamers provided in RevertAid First Strand cDNA Synthesis Kit (Thermo Fisher Scientific Inc., Waltham, MA, USA) followed by SYBR Green-based qPCR reaction (SYBR^®^ FAST qPCR Kit, Kapa Biosystems, Inc., Wilmington, MA, USA). The cycling conditions were as follows: 50 °C for 2 min, 95 °C for 10 min, followed by 40 cycles of 95 °C for 10 sec and 60 °C for 1 min. The end-point used in the real-time quantification was calculated by the StepOne software (v2.2.2, Applied Biosystmes, Carlsbad, CA, USA, 2011), and the threshold cycle number (Ct value) for each analyzed sample was calculated. The primer sets used in this study were listed as followed:

SMURF2
Forward: 5’-TAGCCCTGGCAGACCTCTTA-3’
Reverse: 5’- AATACACCTGGCCTTGTTGC-3’
MRPL19 (internal control)
Forward: 5’- GGGATTTGCATTCAGAGATCAG-3’
Reverse: 5’- GGAAGGGCATCTCGTAAG-3’
qPCR data were analyzed as previous described [35].

### 5.8. Statistical Analysis

Quantitative data were presented as the mean ± SD. The comparisons between two groups were analyzed with Student’s *t*-test. The comparisons among multiple groups (more than three) were analyzed with one-way ANOVA and performed post-hoc test with Tukey Multiple Comparison analysis. A *p*-value of less that 0.05 was considered significantly different.

## Supplementary Materials

The following are available online at www.mdpi.com/2072-6651/8/5/127/s1, Figure S1: Ovatodiolide did not inhibit Hsp27 mRNA expression in BCSCs. BCSCs were enriched by primary mammosphere cultivation from AS-B145 or BT-474 breast cancer cells and dissociated into single cell suspension followed by treated with ovatodilide (1 or 4 mM) or 0.1% DMSO for 48 h. Total RNA were than extracted and the Hsp27 mRNA expression was determined by SYBR Green-based qRT-PCR method.

## Abbreviations

The following abbreviations are used in this manuscript:

CSCs	Cancer stem/progenitor cells
Hsp27	heat shock protein 27
Ova	Ovatiodiolide
Oct4	POU class 5 homeobox 1
Nanog	Nanog homeobox
SMURF2	SMAD ubiquitin regulatory factor 2
