# Supplementary material for: Ovatodiolide Inhibits Breast Cancer Stem/Progenitor Cells through SMURF2-Mediated Downregulation of Hsp27
Source: Toxins (Basel). 2016 Apr 28;8(5):127. doi: 10.3390/toxins8050127 (PMC4885042; doi:10.3390/toxins8050127)
Supplement: Supplementary file 1 [file toxins-08-00127-s001.pdf]

Supplementary Materials: Ovatodiolide Inhibits Breast Cancer Stem/Progenitor Cells through SMURF2-Mediated Downregulation of Hsp27

Kuan-Ta Lu, Bing-Yen Wang, Wan-Yu Chi, Ju Chang-Chien, Jiann-Jou Yang, Hsueh-Te Lee, Yew-Min Tzeng and Wen-Wei Chang

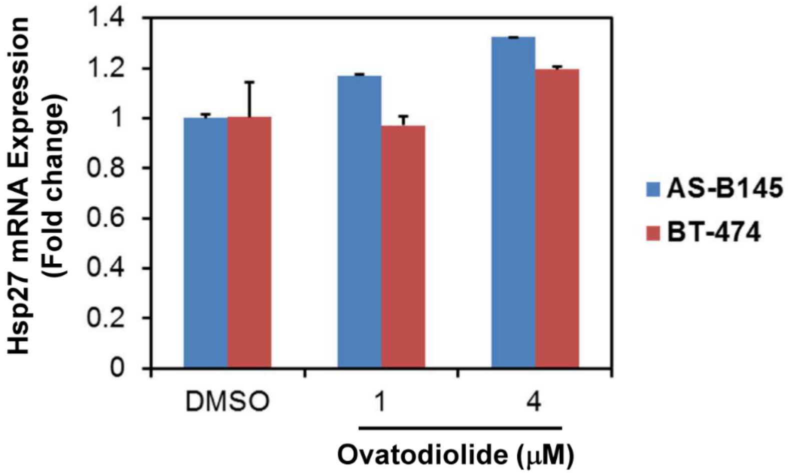

Figure S1. Ovatodiolide did not inhibit Hsp27 mRNA expression in BCSCs. BCSCs were enriched by primary mammosphere cultivation from AS-B145 or BT-474 breast cancer cells and dissociated into single cell suspension followed by treated with ovatodilide (1 or 4 μM) or 0.1% DMSO for 48 h. Total RNA were than extracted and the Hsp27 mRNA expression was determined by SYBR Green-based qRT-PCR method.
